# Leg Spasticity and Ambulation in Multiple Sclerosis

**DOI:** 10.1155/2014/649390

**Published:** 2014-06-04

**Authors:** Swathi Balantrapu, Jacob J. Sosnoff, John H. Pula, Brian M. Sandroff, Robert W. Motl

**Affiliations:** ^1^Department of Kinesiology and Community Health, University of Illinois at Urbana-Champaign, 231 Freer Hall, 906 South Goodwin Avenue, Urbana, IL 61801, USA; ^2^Department of Neurology, NorthShore University HealthSystem, 2650 Ridge Avenue, Evanston, IL 60201, USA

## Abstract

*Background*. Spasticity of the legs is common in multiple sclerosis (MS), but there has been limited research examining its association with ambulatory outcomes. *Objective*. This study examined spasticity of the legs and its association with multiple measures of ambulation in persons with MS. *Methods*. The sample included 84 patients with MS. Spasticity of the legs was measured using a 5-point rating scale ranging between 0 (normal) and 4 (contracted). Patients completed the 6-minute walk (6 MW), timed 25 foot walk (T25FW), and timed up-and-go (TUG), and O_2_ cost of walking was measured during the 6 MW. The patients undertook two walking trials on a GAITRite (CIR systems, Inc.) for measuring spatial and temporal parameters of gait. The patients completed the Multiple Sclerosis Walking Scale-12 (MSWS-12) and wore an accelerometer over a seven-day period. *Results*. 52% (*n* = 44) of the sample presented with spasticity of the legs. Those with leg spasticity had significantly worse ambulation as measured by 6 MW (*P* = 0.0001, *d* = −0.86), T25FW (*P* = 0.003, *d* = 0.72), TUG (*P* = 0.001, *d* = 0.84), MSWS-12 (*P* = 0.0001, *d* = 1.09), O_2_ cost of walking (*P* = 0.001, *d* = 0.75), average steps/day (*P* < 0.05, *d* = −0.45), and walking velocity (*P* < 0.05, *d* = −0.53) and cadence (*P* < 0.05, *d* = −0.46). *Conclusion*. Leg spasticity was associated with impairments in ambulation, including alterations in spatiotemporal parameters and free-living walking.

## 1. Introduction

Spasticity is a common symptom of MS that has a putative negative effect on ambulation. Data from the Patient Registry of the North American Research Committee on Multiple Sclerosis indicated that 84% of persons with MS reported spasticity [1]. Spasticity is most common in the leg muscles indicating that it might be associated with walking. The presence of spasticity in the plantar flexors based on the Modified Ashworth Scale has been associated with worse walking performance based on the timed 25 foot walk (T25FW), timed up-and-go (TUG), and 6-minute walk (6 MW) as well as worse perceived walking impairment based on the Multiple Sclerosis Walking Scale-12 (MSWS-12) scores in persons with MS [2]. The presence of spasticity in the plantar flexors further has been associated with an elevated oxygen (O_2_) cost of walking on a treadmill as a physiological measure of walking in persons with MS [3]. To date, there is limited information on the severity of spasticity in the legs and its association with walking in MS.

We further are unaware of research that has examined the associations among spasticity, spatial and temporal parameters of gait, and free-living ambulation in persons with MS. The evaluation of time and distance parameters during walking can be undertaken using an instrumented walkway [4, 5] and is important for understanding possible mechanisms of the association between spasticity and indicators of walking performance in persons with MS. The evaluation of free-living ambulation can be undertaken objectively based on accelerometry [6, 7] and is important for documenting the possible association of spasticity and walking beyond the laboratory setting and into community living. Collectively, the focus on these new outcomes would provide (a) evidence for why spasticity might be associated with walking performance based on gait kinematics and (b) evidence for spasticity and walking in an ecologically valid context (i.e., the real world).

This study involved a comprehensive examination of the association between spasticity of the leg muscles and walking outcomes in persons with MS. We first replicated and confirmed previous research by examining the association between the presence of spasticity in the leg muscles and performance (T25FW, TUG, and 6 MW), physiological (O_2_ cost of walking), and perceived (MSWS-12) measures of walking; the replication was important for confirming previous research in the context of the measures, particularly spasticity, and sample in the present study. We then extended previous research by examining the association between the presence of spasticity in the leg muscles and kinematic (GAITRite instrumented walkway) as well as community (accelerometry) measures of walking. We further examined the association between severity of spasticity in the leg muscles and the comprehensive battery of walking outcomes. Consistent with previous research [2, 3], we hypothesized that persons with MS who have spasticity of the leg muscles would walk a shorter distance but expend greater energy during 6 MW, take longer to complete the T25FW and TUG, and report higher scores on MSWS-12. We further hypothesized that persons with MS who have spasticity of the leg muscles would have reduced velocity, cadence, single leg support, and increased base of support, step time, and double leg support as well as reduced community ambulation measured as steps per day by accelerometer. We lastly expected that the severity of spasticity would be associated with greater walking dysfunction in MS.

## 2. Methods

### 2.1. Participants

The sample consisted of 84 patients with a clinically definite diagnosis of MS who were recruited through the practices of three locally residing neurologists. The five criteria for inclusion were (a) capacity for independent ambulation or ambulation with an assistive device including cane, crutch, walking frame, or rollator walker; (b) age between 18 and 65 years; (c) willingness to undergo testing; (d) relapse free during the previous 30-day period before testing; and (e) absence of orthopedic and other neurological disorders; we did not screen for diabetic neuropathy or vestibular problems. The mean ± standard deviation age of the sample was 50 ± 10 years and the sample was predominantly female (*n* = 68 or 81% women/*n* = 16 or 19% men). The sample primarily had a relapsing-remitting clinical course (*n* = 69 or 82% of cases) and the mean ± standard deviation disease duration was 11 ± 9 years. Of the 84 persons, 85% or 71 participants reported taking a disease-modifying therapy. The median Expanded Disability Status Scale (EDSS) [8] score, included for descriptive purposes and performed by the same clinician throughout the study, was 4.5 with an interquartile range (IQR) of 3.0 and 6.0. Out of 84 participants, only 34% or 29 participants used an assistive device and 66% or 55 participants did not use an assistive device during the ambulatory tests.

### 2.2. Primary Measures

#### 2.2.1. Spasticity

Spasticity of the legs was measured by a single neurologist throughout the study as a part of the neurological examination for generating an EDSS score [8]. The neurologist was certified by American Board of Psychiatry and Neurology and certified and trained by Neurostatus. Neurostatus is an independent Internet platform for training and certification of physicians participating in clinical research that uses a standardized, quantified neurological examination and assessment of Kurtze's Functional Systems and EDSS scores in persons with multiple sclerosis. This is important for generating valid and reliable data using the Neurostatus examination and Kurtzke's Functional Systems and the EDSS. The neurologist was uninvolved in the mobility assessments, and those who assessed walking mobility had no involvement in spasticity evaluation (i.e., minimum bias with the subjective evaluation of spasticity). Spasticity was measured by passively moving each of the legs through the available range of motion for specific muscle groups including hip flexors, knee extensors, and plantar flexors. The spasticity of the specific muscle groups was rated using a score that ranged from 0 to 4, where 0 = normal; 1 = mild, barely increased muscle tone; 2 = moderate, moderately increased muscle tone that can be overcome and full range of motion is possible; 3 = severe, severely increased muscle tone that is extremely difficult to overcome and full range of motion is not possible; and 4 = contracted. The spasticity assessment within the neurological examine for the EDSS is consistent with the Modified Ashworth Scale (MAS) as both measures have similar descriptors for scoring. The total spasticity score for both the legs was calculated by summing the individual spasticity scores for the right and left legs. The data analyses used the overall severity score and categorized persons into groups of “spasticity” (total spasticity score other than zero) and “no spasticity” (total spasticity score is zero), consistent with previous research [2].

#### 2.2.2. 6 MW

The 6 MW was performed in a rectangular, carpeted corridor with hallways that exceed 50 m in length and that was clear of obstructions and foot traffic. We provided standardized instructions and emphasized walking as far and as fast as possible for 6 minutes [9]. One researcher followed alongside of the participant for safety, while another researcher followed 1 meter behind the participant and recorded the distance travelled in feet using a measuring wheel (Stanley MW50, New Briton, CT) [10].

#### 2.2.3. T25FW

T25FW is an indicator of ambulatory impairment and is a component of the multiple sclerosis functional composite (MSFC) [11, 12]. The T25FW was performed along a clearly marked 25-foot long path in a carpeted corridor that was clear of obstructions and foot traffic and had a hand rail for safety. We provided standardized instructions and emphasized walking as fast and as safely as possible [11]. One researcher followed alongside of the participant for safety and another recorded the time in seconds by using a stopwatch. An average of the two trials was included for the analysis.

#### 2.2.4. TUG

The TUG was performed in a patient room and on a carpeted floor with a clearly marked 10-foot distance from a stabilized chair. Participants were given standardized instructions, namely, to stand up from the chair without using arms to push off from the armrests of the chair, walk for 10 feet as fast and safely as possible, return to the chair, and sit down. The test has been validated previously for use in MS [13]. The time was recorded in seconds by using a stopwatch, and two trials were conducted and an average of the two was taken for analysis.

#### 2.2.5. MSWS-12

The MSWS-12 scale is a validated 12-item questionnaire designed to assess the perceived walking impairment in an individual with MS during the past two weeks [14]. All the 12 items were rated on an ordinal scale ranging from 1 (not at all) to 5 (extremely). The total MSWS-12 score ranges between 0 and 100 and was computed by summing all items scores, subtracting the minimum possible score (12), dividing the score by 48, and then multiplying the result by 100 [14].

#### 2.2.6. O_2_ Cost of Walking

$\dot{\text{V}}{\text{O}}_{2}$ was measured during the 6 MW using a commercially available portable metabolic unit (K4b^2^ Cosmed, Italy). The O_2_ and CO_2_ analyzers of the portable metabolic unit were calibrated using verified concentrations of gases, and the flow-meter was calibrated using a 3-L syringe (Hans Rudolph, Kansas City, MO). Steady-state $\dot{\text{V}}{\text{O}}_{2}$ was calculated by averaging $\dot{\text{V}}{\text{O}}_{2}$ values across the final 3 minutes (minutes 4–6) of the 6 MW. The O_2_ cost of walking was expressed as mL·kg^−1^·m^−1^ by dividing steady-state $\dot{\text{V}}{\text{O}}_{2}$ in mL·kg^−1^·min^−1^ by actual walking speed in m·min^−1^ [10].

#### 2.2.7. GAITRite

Participants completed 2 walking trials along a 26-foot GAITRite (CIR systems, Inc.) electronic walkway at a comfortable, self-selected pace as done in previous research involving persons with MS [4, 5]. We recorded spatial and temporal parameters of gait including velocity (cm·sec^−1^), cadence (steps·min^−1^), base of support (cm), step time (sec), single leg support (% of gait cycle), double leg support (% of gait cycle), and swing phase (% of gait cycle). The average of the 2 trials for each variable was used in the analysis for improved reliability.

#### 2.2.8. Accelerometry

The participants wore an Actigraph model GT3X accelerometer (Health One Technologies, Fort Walton Beach, FL) on an elastic belt around the waist on the nondominant hip during the waking hours, except while showering, bathing, and swimming, for a 7-day period. Waking hours were defined as the duration from the point of waking out of bed in the morning until the point of going to bed in the evening. The participants were provided with a log sheet to record the time the accelerometer was worn and this was verified by checking the minute-by-minute accelerometer data. We summed the minute-by-minute step counts over each day and then averaged the values over the 7 days (steps·d^−1^). There is evidence that accelerometers and the metric of steps·d^−1^ provide a valid and reliable measure of ambulatory physical activity in the community among persons with MS [6, 7] and healthy adults [15].

### 2.3. Procedure

The procedure was approved by a University Institutional Review Board and all participants provided written informed consent. The measures were administered on standardized time schedule during a single session, with the exception of accelerometry, by the trained and experienced staff of an MS research center. The participants initially provided demographic information, completed the MSWS-12, and then underwent a neurological examination by a single clinician for generating an EDSS score and assessment of leg spasticity. This was followed by performance of the ambulatory measures, namely, 6 MW, T25FW, and TUG, and 2 walking trials on the GAITRite. The participants were then provided with an accelerometer that was worn over a 7-day, free-living period and the device and accompanying log were returned through the U.S. Postal Service in a prestamped, preaddressed envelope. All participants received $15 remuneration towards travel expenses.

### 2.4. Data Analysis

The data analysis was conducted using SPSS version 18 (SPSS Inc., Chicago, IL). We initially compared the groups on demographic and clinical characteristics using independent samples *t*-tests that were parametric (i.e., age, height, weight, and duration of MS) and nonparametric (i.e., EDSS) as well as chi-square statistics (i.e., sex and type of MS). This was followed by an examination of the distributions for data on all ambulatory outcomes based on estimation of skewness and kurtosis values. We then provided descriptive statistics including mean, median, standard deviation (SD), and range of scores for the ambulatory outcomes. We performed inferential statistics for the difference in the ambulatory outcomes between groups with and without leg spasticity by conducting independent samples *t*-tests; we performed nonparametric independent samples *t*-tests using Mann-Whitney *U* for confirming group differences for the *t*-test with outcomes that had nonnormal distributions. We opted for this approach as effect size estimates are derived from mean scores rather than medians and this would logically follow for a parametric analysis on means. The magnitude of group differences was estimated by calculating the Cohen's *d* effect size (i.e., difference in mean scores divided by pooled standard deviation) [16]. Values for Cohen's *d* of 0.2, 0.5, and 0.8 were interpreted as small, moderate, and large, respectively [16]. The ambulatory outcomes included the 6 MWT, T25FW, and TUG performance; MSWS-12 scores; O_2_ cost of walking; spatial and temporal parameters of gait by the GAITRite; and average activity counts per day and step counts per day over 7 days by accelerometer. The alpha for statistical significance was set at 0.05. We did not adjust alpha for the multiple comparisons because of our directional hypotheses and replication of previous research. We further provided Spearman rank-order correlation coefficients (*r*_*s*_) for the association between spasticity severity and walking outcomes. Values for correlations of 0.1, 0.3, and 0.5 were interpreted as small, moderate, and large, respectively [16].

## 3. Results

### 3.1. Demographic and Clinical Differences between Groups

The mean scores, standard deviations, ranges of scores, and the appropriate statistical test for the difference between the groups on demographic and clinical measures are provided in Table 1. Of the 84 participants, 44 or 52% of the sample had spasticity of the legs based on the neurological examination, whereas 40 or 48% of the sample did not have spasticity. The mean and median leg spasticity scores (sum of rating for each muscle) for the group with spasticity were 1.8 (SD = 0.7) and 2.0, respectively. The mean and median leg spasticity scores (sum of rating for each muscle) for the entire sample were 0.9 (SD = 1.1) and 1.0, respectively. The study participants were both men (*n* = 4 without leg spasticity and *n* = 12 with leg spasticity) and women (*n* = 36 without spasticity and *n* = 32 with spasticity) who had a clinically definite diagnosis of MS; there was a significantly larger percentage of men in the sample with leg spasticity (chi-square = 4.05; *P* < 0.05). Relapsing-remitting MS was most common in those without leg spasticity (93% of cases) and those with leg spasticity (81% of cases); there was not a significant difference in MS type between groups (chi-square = 3.75; *P* = 0.15). As would be expected, those with spasticity of the legs had significantly higher EDSS scores than those without spasticity (*Z* = 3.73; *P* < 0.0001); this was driven by the Pyramidal (*Z* = 2.72; *P* < 0.01), Cerebellar (*Z* = 3.41; *P* < 0.001), Sensory (*Z* = 2.92; *P* < 0.005), and Mental (*Z* = 2.84; *P* < 0.005) Functional System scores. There were no statistically significant differences between groups in height, weight, or duration since MS onset, but the group with spasticity was significantly older than those without spasticity (*t* = 2.38; *P* < 0.05).

### 3.2. Ambulatory Outcomes

The estimates of both skewness and kurtosis were less than 2.58 (i.e., 3 SDs) for all ambulatory and gait outcomes, with the exception of T25FW (skewness = 2.62; kurtosis = 11.2), TUG (skewness = 2.38; kurtosis = 8.1), and O_2_ cost of walking (skewness = 2.38; kurtosis = 2.64); we provide both parametric and nonparametric *t*-tests in the text for those three variables when comparing groups of persons with and without spasticity. The mean (standard deviation) and median scores, ranges of scores, effect sizes for the ambulatory outcomes per group with and without leg spasticity are provided in Table 2. The same data are provided for the spatial and temporal parameters of gait per group with and without leg spasticity in Table 3.

#### 3.2.1. Walking Performance

**Figure 1:**
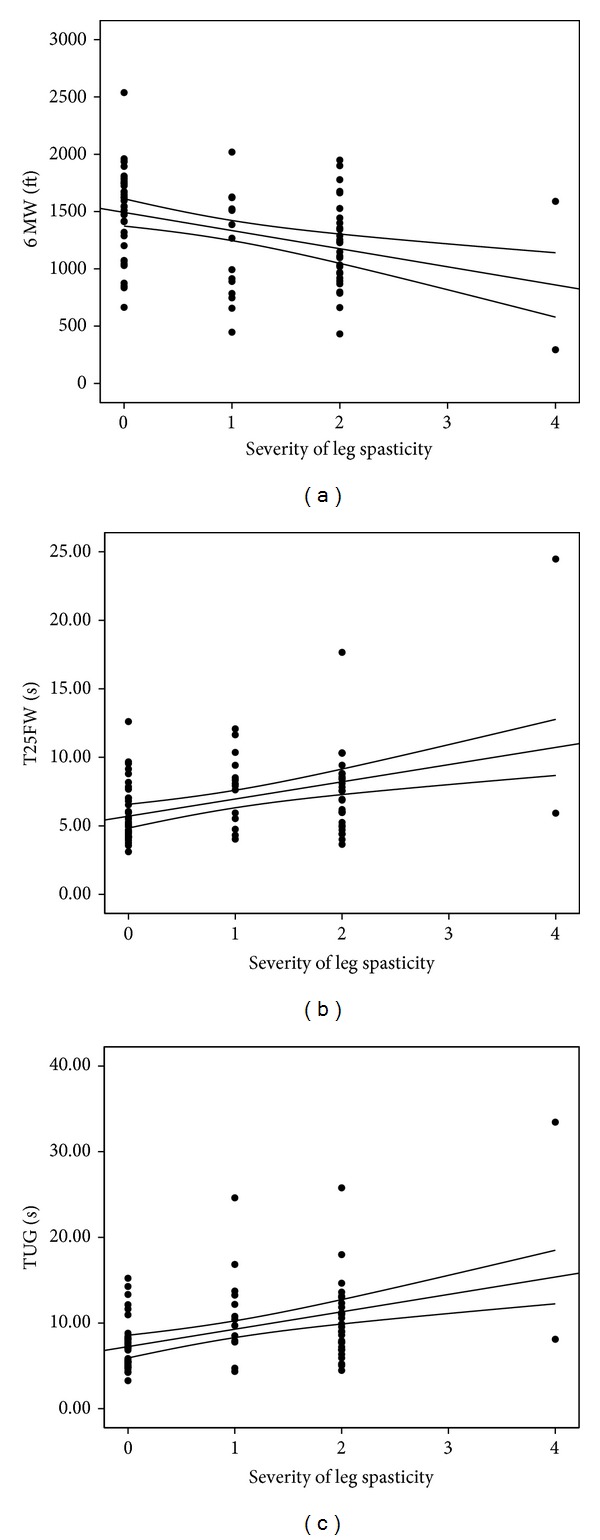
Scatter plots of the association between severity of leg spasticity and walking performance measured by the 6-minute walk (6 MW), time 25 foot walk (T25FW), and timed up-and-go (TUG).

6 MW distance was significantly shorter (*t* = −3.93; *P* < 0.0001; Cohen's *d* = −0.86) in those with leg spasticity compared to those without leg spasticity. T25FW (*t* = 3.11; *P* < 0.005; Cohen's *d* = 0.72; *Z* = 3.25; *P* < 0.001) and TUG (*t* = 3.61; *P* < 0.001; Cohen's *d* = 0.84; *Z* = 3.75; *P* < 0.0001) times were significantly longer for those with leg spasticity compared to those without leg spasticity. There were statistically significant and moderate associations between overall spasticity severity and 6 MW (*P* = 0.001; *r*_*s*_ = −0.37), T25FW (*P* = 0.0001; *r*_*s*_ = 0.40), and TUG (*P* = 0.0001; *r*_*s*_ = 0.42) performance; scatter plots for those associations are provided in Figure 1.

#### 3.2.2. Perceived Walking Impairment

Perceived walking impairment based on MSWS-12 scores was significantly higher (*t* = 4.98; *P* < 0.0001; Cohen's *d* = 1.09) in those with leg spasticity compared to those without leg spasticity. There was a statistically significant and moderate association between overall spasticity scores and MSWS-12 scores (*P* = 0.0001; *r*_*s*_ = 0.41).

#### 3.2.3. O_2_ Cost of Walking

The energetic cost of walking was significantly higher (*t* = 3.20; *P* < 0.001; Cohen's *d* = 0.75; *Z* = 3.41; *P* < 0.001) in those with leg spasticity compared to those without leg spasticity. There was a statistically significant and moderate association between overall spasticity scores and O_2_ cost of walking (*P* = 0.0001; *r*_*s*_ = 0.34).

#### 3.2.4. Free-Living Ambulation

Free-living ambulation based on steps·d^−1^ was significantly lower (*t* = −2.07; *P* < 0.05; Cohen's *d* = −0.45) in those with leg spasticity compared to those without leg spasticity. There was a small, nonstatistically significant association between overall spasticity scores and steps·d^−1^ (*P* = 0.07; *r*_*s*_ = −0.20).

#### 3.2.5. Spatial and Temporal Parameters of Gait

Velocity (*t* = −2.44; *P* < 0.05; Cohen's *d* = −0.54) and cadence (*t* = −2.10; *P* < 0.05; Cohen's *d* = −0.46) were significantly slower for those with leg spasticity compared to those without leg spasticity. The differences approached significance for single leg support (*t* = −1.88; *P* = 0.07; Cohen's *d* = −0.41), double leg support (*t* = 1.81; *P* = 0.07; Cohen's *d* = −0.40), and step time (*t* = 1.68; *P* = 0.10; Cohen's *d* = 0.37) but not base of support (*t* = 1.01; *P* = 0.32; Cohen's *d* = 0.22). There were small-to-moderate, statistically significant associations between overall spasticity scores and walking velocity (*P* < 0.01; *r*_*s*_ = −0.30) and single (*P* < 0.01; *r*_*s*_ = −0.28) and double support (*P* < 0.01; *r*_*s*_ = 0.28); the associations were small and not statistically significant with cadence (*P* = 0.24; *r*_*s*_ = −0.13), base of support (*P* = 0.11; *r*_*s*_ = 0.18), and step time (*P* = 0.44; *r*_*s*_ = 0.09).

## 4. Discussion

This study demonstrated that those with leg spasticity had worse performance on ambulatory outcomes including the 6 MW, T25FW, and TUG; scored higher on the MSWS-12; had elevated O_2_ cost of walking; had altered velocity and cadence as spatial and temporal parameters of gait; and had reduced free-living ambulation compared to those without leg spasticity. The severity of spasticity mattered for 6 MW, T25FW, TUG, MSWS-12, O_2_ cost of walking, walking velocity, and single and double support. Collectively, these findings highlight that leg spasticity is associated with a comprehensive abnormal pattern of ambulation that extends from the laboratory into real life, and this highlights the importance of optimizing spasticity through its management for maintaining and perhaps improving mobility in persons with MS. This recognizes that some degree of spasticity might be important for walking in some cases of MS, whereas spasticity might be detrimental for walking in other cases of MS. This might be an important avenue of future research on optimizing spasticity for maximizing mobility in MS, particularly given that spasticity can represent a compensative mechanism for weakness that is associated with walking impairment in MS.

There are important limitations of the current study. One limitation is that spasticity alone might not completely account for altered mobility outcomes in persons with MS and we did not measure other contributing factors such as muscle strength, proprioception, and range of motion in this study [6]. We were interested in spasticity and mobility and could have measured other variables but opted against this considering constraints of time and number of assessments as well as the number of personnel available for block testing. The addition of a strength assessment, for example, would have provided important information but is ideally measured using dynamometry and this requires lengthy protocols and might induce unnecessary fatigue. The second limitation is that we measured spasticity as part of a neurological examination for generating EDSS scores rather than using a clinical outcome such as the MAS. This creates a problem of not being able to control EDSS scores in the analyses as the measure of spasticity is part of the EDSS (i.e., the measures are not independent). Thirdly, we had minimal exclusion criteria in the selection of the sample because we wanted to be representative of MS population, but other factors such as diabetic neuropathy, urinary tract infections, medications, or vestibular problems might account for differences in ambulatory outcomes. Fourth, the inclusion of persons who use an assistive device and/or antispastic medications might have altered the ambulatory measures. For example, there is not a significant difference between the group with and the group without spasticity in terms of base of support and this might be attributed to assistive devices used by persons with MS. Lastly, this study is limited by a lack of information on location of spasticity within the limb and the association with ambulatory outcomes, but the focus was on identifying the overall impact on ambulation and not location of spasticity. Despite those limitations, this study provides evidence regarding the pervasive association between spasticity on mobility outcomes in MS and highlights the importance of identifying and evaluating interventions that can optimize spasticity with potential beneficial effects on laboratory and real-life mobility outcomes.

The results of this study both replicate and extend previous research on spasticity and mobility outcomes in MS. For example, previous studies have reported that the presence of leg spasticity is associated with 6 MW, T25FW, and TUG performance, MSWS-12 scores, and O_2_ cost of walking in persons with MS [2, 3, 17]; we now confirm and replicate those results. Importantly, the O_2_ cost of walking was previously measured on a treadmill [3] and we now replicate and extend the association between spasticity and energetic cost of walking measured during over-ground walking in persons with MS. Moreover, we further extended previous research by indicating that leg spasticity is associated with alterations in velocity and cadence as spatiotemporal parameters of gait and steps·d^−1^ as a marker of community ambulation in persons with MS. Although speculative, spasticity might affect velocity and cadence because of increased resistance to the movement of the lower extremities, and it might have affected community ambulation because of effects on walking capacity (6 MW) and energetics (O_2_ cost of walking) resulting in changes in perceived (MSWS-12) and actual walking impairment in daily life. We further report on the severity of spasticity and its association with markers of walking dysfunction. Collectively, the breadth of possible alterations in ambulation, particularly the novel associations with spatial and temporal parameters of gait and community ambulation, underscores the importance of designing therapeutic strategies for optimizing spasticity and testing the effect for improving mobility in MS. Such interventions might target spasticity of the legs for improving spatial and temporal parameters of gait with downstream effects on performance and self-reported, physiological, and free-living measures of ambulation.

Overall, we report that leg spasticity was present in more than half of the persons with MS and was associated with impairments in ambulation, including alterations in spatial and temporal parameters and free-living walking. This pervasive association between spasticity of the leg and ambulation, from the gait cycle through community walking, further highlights the importance of designing and testing interventional therapeutics for spasticity management and possible consequences for mobility in MS.

## Figures and Tables

**Table 1:** Mean, standard deviation, and range of values for demographic and clinical variables for the sample of persons who have multiple sclerosis with (*n* = 44) and without (*n* = 40) leg spasticity.

Variable	Group	Mean (SD)	Range	Statistic	*P* value
Age (years)	No spasticity	47.7 (11.1)	30–64	2.38	<0.05
	Spasticity	52.8 (7.6)	30–65		

Height (cm)	No spasticity	167.8 (8.6)	157–187	1.03	ns
	Spasticity	169.8 (9.3)	156–193		

Weight (kg)	No spasticity	79.0 (17.2)	53–118	1.21	ns
	Spasticity	84.5 (23.5)	46–138		

MS duration	No spasticity	11.5 (9.7)	0–39	0.57	ns
	Spasticity	10.3 (8.4)	0–39		

EDSS (mdn)	No spasticity	3.5	2.0–6.5	3.73	<0.0001
	Spasticity	6.0	2.5–6.5		

Note: SD: standard deviation; mdn: median, ns: nonsignificant. Statistic represents value of parametric independent samples *t*-test, with exception of EDSS that is nonparametric.

**Table 2:** Descriptive and effect size data for differences in ambulatory outcomes between the samples of persons who have multiple sclerosis with (*n* = 44) and without (*n* = 40) leg spasticity.

Variable	Group	Mean (SD)	Median	Range	SD pooled	Cohen's *d*	Statistic	*P* value
6MW (ft)	No spasticity	1525 (379)	1623	664–2538	398	−0.86	−3.93	<0.0001
	Spasticity	1182 (417)	1188	294–2019				

T25FW (sec)	No spasticity	5.8 (2.1)	5.0	3.1–12.6	2.9	0.72	3.11	<0.005
	Spasticity	7.9 (3.7)	7.6	3.7–24.5				

TUG (sec)	No spasticity	7.2 (2.8)	6.9	3.3–15.2	4.4	0.84	3.61	<0.001
	Spasticity	10.9 (5.7)	9.6	4.4–33.5				

MSWS-12	No spasticity	31.3 (26.9)	30.2	0–85.4	24.4	1.09	4.98	<0.0001
	Spasticity	58.0 (22.1)	62.5	14.6–93.8				

O_2_ Cost (mL·kg^−1^·m^−1^)	No spasticity	0.197 (0.040)	0.186	0.143–0.317	0.063	0.75	3.20	<0.001
	Spasticity	0.244 (0.083)	0.224	0.137–0.556				

Accelerometry (steps·d^−1^)	No spasticity	4708 (2747)	4376	630–13136	2437	−0.45	−2.07	<0.05
	Spasticity	3601 (2155)	3089	419–8178				

Note: SD: standard deviation; 6MW: 6-minute walk; T25FW: timed 25 foot walk; TUG: timed up-and-go; MSWS-12: Multiple Sclerosis Walking Scale-12; O_2 _cost: oxygen cost of walking.

**Table 3:** Descriptive and effect size data for differences in spatial and temporal parameters of gait between the samples of persons who have multiple sclerosis with (*n* = 44) and without (*n* = 40) leg spasticity.

Variable	Group	Mean (SD)	Median	Range	SD pooled	Cohen's *d*	Statistic	*P* value
Velocity (cm·sec^−1^)	No spasticity	112.8 (30.2)	107.8	59.6–172.1	30.1	−0.53	−2.44	<0.05
	Spasticity	96.6 (30.0)	95.5	30.6–145.7				

Cadence (steps·min^−1^)	No spasticity	108.9 (14.8)	110.1	74.6–145.2	14.9	−0.46	−2.11	<0.05
	Spasticity	102.0 (14.9)	104.4	74.3–133.9				

Base of support (cm)	No spasticity	12.3 (4.3)	11.8	2.42–23.89	4.4	0.22	1.01	ns
	Spasticity	13.3 (4.4)	12.6	5.1–21.8				

Step time (sec)	No spasticity	0.58 (0.09)	0.56	0.41–0.82	0.09	0.37	1.68	ns
	Spasticity	0.62 (0.10)	0.59	0.47–0.82				

Single leg support (%)	No spasticity	34.5 (2.7)	34.5	27.7–38.7	2.72	−0.41	−1.88	ns
	Spasticity	33.4 (2.8)	33.8	26.1–39.4				

Double leg support (%)	No spasticity	31.2 (5.3)	31.4	23.2–44.8	5.5	0.40	1.81	ns
	Spasticity	33.4 (5.6)	32.7	21.1–48.0				

Note: SD: standard deviation; ns: nonsignificant.
